# Enhancing maturity in 3D kidney micro‐tissues through clonogenic cell combinations and endothelial integration

**DOI:** 10.1111/jcmm.18453

**Published:** 2024-05-31

**Authors:** Fatemeh Abdollahzadeh, Niloofar Khoshdel‐Rad, Khadijeh Bahrehbar, Saiedeh Erfanian, Vahid Ezzatizadeh, Mehdi Totonchi, Reza Moghadasali

**Affiliations:** ^1^ Department of Stem Cells and Developmental Biology, Cell Science Research Center Royan Institute for Stem Cell Biology and Technology, ACECR Tehran Iran; ^2^ Department of Developmental Biology University of Science and Culture Tehran Iran; ^3^ Medical Genetics Department Ayandeh Clinical and Genetic Laboratory Varamin Iran

**Keywords:** clonogenic epithelial‐like cells, clonogenic mesenchymal‐like cells, co‐culture, kidney micro‐tissues

## Abstract

As an advance laboratory model, three‐dimensional (3D) organoid culture has recently been recruited to study development, physiology and abnormality of kidney tissue. Micro‐tissues derived from primary renal cells are composed of 3D epithelial structures representing the main characteristics of original tissue. In this research, we presented a simple method to isolate mouse renal clonogenic mesenchymal (MLCs) and epithelial‐like cells (ELCs). Then we have done a full characterization of MLCs using flow cytometry for surface markers which showed that more than 93% of cells expressed these markers (Cd44, Cd73 and Cd105). Epithelial and stem/progenitor cell markers characterization also performed for ELC cells and upregulating of these markers observed while mesenchymal markers expression levels were not significantly increased in ELCs. Each of these cells were cultured either alone (ME) or in combination with human umbilical vein endothelial cells (HUVECs) (MEH; with an approximate ratio of 10:5:2) to generate more mature kidney structures. Analysis of 3D MEH renal micro‐tissues (MEHRMs) indicated a significant increase in renal‐specific gene expression including *Aqp1* (proximal tubule), *Cdh1* (distal tubule), *Umod* (loop of Henle), *Wt1*, *Podxl* and *Nphs1* (podocyte markers), compared to those groups without endothelial cells, suggesting greater maturity of the former tissue. Furthermore, ex ovo transplantation showed greater maturation in the constructed 3D kidney.

## 1 INTRODUCTION

Kidney diseases pose a significant global health concern, affecting over 15% of the world's population.^1^ In mammals, the kidneys serve crucial homeostatic functions, regulating pH, electrolytes and fluid balance, while also playing pivotal endocrine roles through the secretion of hormones such as erythropoietin (EPO), components of the renin‐angiotensin system (RAS), calcitriol and prostaglandins.^2^ The loss of tubular epithelial cells (TECs) due to acute or chronic injuries can lead to renal failure,^3^, ^4^ emphasizing the importance of replacing necrotic tubular cells to restore renal function partially. Evidence suggests that the proper functioning and regeneration of TECs are essential for maintaining kidney health, whereas impaired epithelial regeneration can contribute to chronic kidney disease (CKD) and the development of tubule‐interstitial renal scarring, ultimately leading to conditions like acute kidney injury (AKI) or fibrosis.^4^ Various supporting cell types, including stromal and endothelial lineages, play crucial roles in renal tissue function.^5^ MSCs possess immune‐evasive properties, allowing them to be transplanted without the need for immunosuppression. Following kidney injury, MSCs have demonstrated the ability to secrete bioactive substances like cytokines/chemokines and growth factors. Rather than directly replacing damaged renal epithelial cells, MSCs influence renal regenerative processes, ultimately expediting the recovery period.^6^ Endothelial cells (ECs) are vital for oxygen and nutrient transport within kidney tissue, supporting kidney physiology and contributing to disease development.^7^, ^8^ Understanding the characteristics and capabilities of major cell populations, including TECs, mesenchymal cells and ECs, is essential for elucidating kidney development mechanisms and potential diseases.

As an approach, cell culture could be used to study potential intracellular mechanisms, treatments, genetic manipulations, drug screening and toxicity of renal cells.^9^ Native physiological conditions and cellular interactions, within the complex heterogeneous tissues, are not generally recapitulated by the conventional two‐dimensional (2D) models. Due to the limitations of 2D cellular models, scientists currently consider the alternative three‐dimensional (3D) methods to study cells in vitro. For instance, cell–cell and cells‐ECM interactions are generally increased by multi‐cellular structures and specific polarity of 3D models.^10^, ^11^ Renal micro‐tissues derived from primary renal cells have emerged as valuable research models for developmental studies, disease modelling and drug screening, recapitulating key features of the original tissue.^12^ However, the roles of adult renal stem cells, including organ‐specific progenitor cells, remain incompletely characterized. Recent evidences showed that the organ‐specific progenitor cells obtained from embryonic rat metanephric mesenchymal cells could differentiate into epithelial, myofibroblasts and smooth muscle cells.^13^ Human resident renal stem cell population, such as Cd133 cells, can contribute to improve renal injury and remind the multipotent properties. In spite of limiting to the self‐renewal capacity, these cells are capable to convert endothelial or TECs into the respectively epithelial and blood cells.^14^ Thus far, isolation of primary renal tubular cells has been performed from several models, including mice,^15^, ^16^ rats,^17^, ^18^, ^19^ canines,^20^ rabbits^21^, ^22^ and humans.^23^ Tubular and glomerular micro‐tissue cells are suitable models to investigate potential mechanisms underlying the normal adult kidney tissue, particular disease models and cell toxicity.^11^ In this regard, objective of the present study was to generate an efficient, simple and economic method to generate 3D renal‐based structure micro‐tissue from epithelial‐like cells (ELCs) and mesenchymal‐like cells (MLCs) obtained from mouse kidney, upon isolating and characterization of them. For that, we selected two groups of gene panels, including *Pax‐8*, *Lgr‐4*, *Cd‐133*, *Sox‐9* for stem/ELCs, and *Cd‐44*, *Cd‐73* and *Cd‐105* for MLCs. After expanding, adult mouse renal cells could generate 3D kidney micro‐tissue in vitro. It was subsequently analysed in terms of self‐renewal. ECs have been demonstrated to be crucial for reserving oxygen and micronutrients as well as transmission of paracrine signals to the other cells in the nephrogenic niche. They can also support kidney organogenesis. Co‐culture of these clonogenic renal cells with endothelial cells (MEH) was utilized, in the current study, to recapitulate the original micro‐tissue features in vitro. Findings showed that MEH renal micro‐tissues (MEHRMs) enhanced gene expression of different renal‐specific markers in ultra‐low attachment (ULA) 96‐well plates.

## 2 MATERIALS AND METHODS

### 2.1 Isolation and culture of renal clonogenic mesenchymal and epithelial‐like cells from mouse kidneys

**FIGURE 1:**
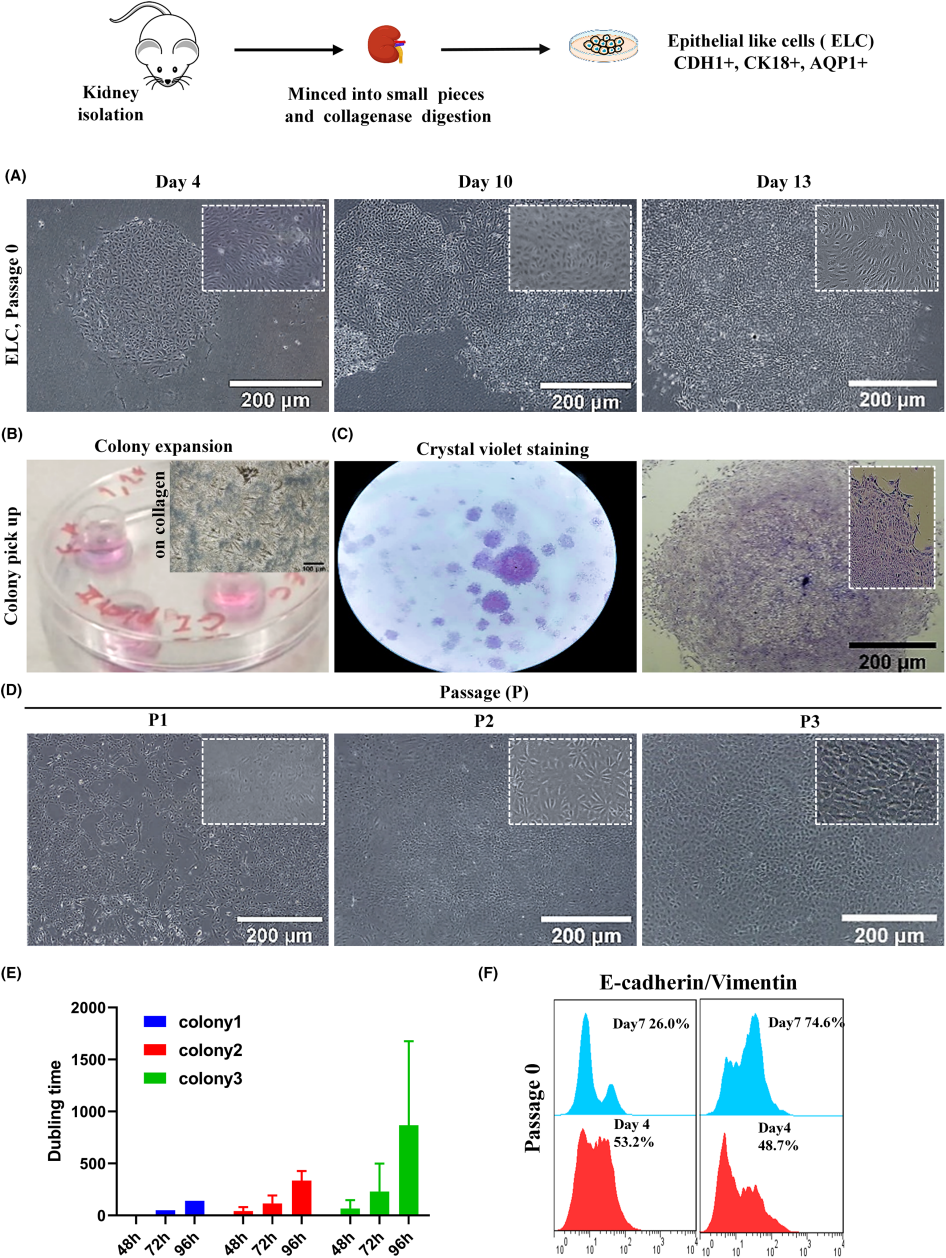
Colony assay, pick up and expansion. (A) Epithelial like cells colony (ELCs), passage 0 (Days 4, 10 and 13). (B) ELCs pick up (by cloning cylinders, passage 0) and expansion on collagen extra cellular matrix (ECM), (C) Colony formation assay at 1 × 10^5^ cells in the 60 mm^3^ culture dish, passage 3 (crystal violet staining). (D) Epithelial like cells colony (ELCs), passage 1‐3. (E) Colony cell doubling time calculation, passage 3 (48, 72 and 96 hours). (F) ELCs flow‐cytometry analysis, at 2 × 10^6^ cells, passage 0 (E‐cadherin and Vimentin expressions, Days 4 and 7).

Approximately 15 FVB male mice were obtained from Royan animal laboratory (Royan Institute, Iran), aging 4–6 weeks old and 15 ± 2 g. Animal cares and procedures were carried out in accordance with the Medical Ethics Committee of Royan Institute protocols (Approval code: IR. ACECR. ROYAN. REC.1397.270). The mice were subsequently sedated using an intraperitoneal injection consisting of 75 mg/kg ketamine (Alfasan) and 0.1 mg/kg medetomidine (Syva). After anaesthetising and sacrificing animals, kidney tissues were collected for the experiments. A perfusion pump at 32 mL/min was utilized, through which the buffer perfusion (phosphate‐buffered saline [PBS]; Thermo Fisher Scientific, USA) supplemented with penicillin–streptomycin 2X was entered to the left ventricle, from the heart apex, using a 27‐G needle. In order to completely empty the body blood and consequently make a pale and bloodless kidney, after PBS perfusion, the right heart atrium was punched, based on the previously established protocol.^4^ Upon leaving the isolated kidneys in sterile petri‐dishes, they were thoroughly cleaned, followed by mincing them into small pieces to prepare a homogenous mixture. Next, quick enzymatic digestion was performed by incubating the minced tissues with 2 mg/mL collagenase type II (Invitrogen, USA) for 10 min at 37°C. To obtain a single‐cell solution, the digested tissue was then gradually passed through a 100 μm cell strainer (Figure 1). To culture the suspended cells, RPMI 1640 (Gibco, USA) was utilized, while it was supplemented with 10% fetal bovine serum (FBS; Gibco, USA), 20 ng/mL epidermal growth factor (EGF; TermoFisher Scientific, USA), 1% penicillin–streptomycin (Gibco, USA), 1% L‐glutamine (Sigma‐Aldrich, USA) and 0.1% β‐mercaptoethanol (Sigma‐Aldrich, USA). The cells were then incubated at 37°C, 95% humidity and 5% CO_2_. They were next detached using specialized cloning cylinders after 7–8 days, upon reaching 80% confluency, by injecting roughly 0.2 mL of 0.25% trypsin (Gibco, USA). The cells were subsequently incubated at 37°C in a humid atmosphere with 5% CO_2_. Ultimately, the cells were examined in terms of morphology, capacity of proliferation and gene expression. In this study, few cell colonies were selected to assess population doubling time (PDT) after 48, 76 and 96 h culture using the bellow formula:

PDT=CT/Log N/N0×3/33

In this study, we implemented the population doubling time (PDT) formula derived from the standard exponential growth equation, where the doubling time (*t*) signifies the duration for a population to double in magnitude. The PDT formula utilized in our analysis is articulated as follows:

$$\mathrm{PDT}=\frac{0.3}{\text{Growthrate}}\times \mathrm{Log}\left(2\right)$$

Here, the inclusion of the 0.3 factor serves as a normalization term originating from the conversion of time units. In our specific research context, this term is introduced to standardize the time unit such that the resultant doubling time is expressed in days. The selection of 0.3 is grounded on the approximation of 1 month being tantamount to 30 days, thereby 3/33 is employed to appropriately scale the time units. To expound further, the growth rate (Gr) is conventionally represented in logarithmic form as Log NtN0, where Nt denotes the final population size, N0 denotes the initial population size, and *t* denotes the time interval. By integrating this logarithmic Gr and the adjustment factor, we ensure the accurate computation of the doubling time in terms of days.

To create ECM for the cultivation of ELCs, white bundles of Type I collagen were obtained from rat tail tendons and dissolved in 0.05 M acetic acid on a magnetic stirrer overnight at 4°C.

### 2.2 Colony‐forming units

Colony formation assay was performed to test renal tissue‐derived clonogenic cell activity using crystal violet staining method. In this regard, 1 × 10^5^ cells were collected from the third passage and seeded in the 60 mm^3^ culture dish. After 7 days culture, the cells created several colonies. The colonies were stained with crystal violet (Sigma, USA) for 10 min after preserving the cells with 10% methanol. Subsequent to the staining, number of colonies was counted using the inverted phase‐contrast microscope (Olympus, Japan). An objective tiny micrometre was used to measure size of the colonies. Colony‐forming unit (CFU) assay was performed for five times using 1 × 10^5^ cells. The cells were seeded in 60 mm^3^ dish and cultured for 14 days to form colonies.

### 2.3 Growth curve

In order to plot growth curve, it is necessary to daily observe the cells and compare their quantities. Therefore, different colonies were cultivated per well on 24‐well plates, and they were cultured for 48, 72 and 96 h. Subsequently, some wells were trypsinized every day and cell counting was performed by using a haemocytometer. GraphPad Prism (version 8.0) was used to illustrate growth curve data.

### 2.4 Multi‐lineage differentiation of mesenchymal‐like cells

MLCs were cultured about 300000 cells in the individual well of the 6‐well from three passages using DMEM medium containing 10% FBS. Upon obtaining 80% confluence, proliferation environment was replaced with osteogenic (i.e. DMEM) medium supplemented with 50 μg/mL ascorbic 2‐phosphate, 10 nM dexamethasone and 10 mM ß‐glycerole phosphate (all from Sigma‐Aldrich, USA). Adipogenic or oseteogenic differentiation culture medium was used for 21 days; meanwhile the culture medium was weekly changed twice. The cells were subsequently stained with Alizarin red to examine osteogenesis. To assess adipose convert potential, the third passage of the cells were cultivated on a 6‐well culture plate. Once the cells reached to 80% confluence, the medium was changed into the differentiation medium containing 100 nM dexamethasone and 50 g/mL indomethacin (both from Sigma‐Aldrich, USA). Finally, the culture medium was weekly replaced twice. In terms of morphology, cell conversion was detected by using oil red staining after 21 days of incubation.

### 2.5 Segregating and plating HUVECs derived from human umbilical cords

Human umbilical vein endothelial cells (HUVECs) were isolated from four donated human umbilical cords, using the same technique as previously described.^24^ In this regard, a formal written consent was obtained from the parents, before birth of the healthy term neonates. In this procedure, the cord was briefly washed with Dulbecco's phosphate‐buffered saline (DPBS; Thermo Fisher Scientific, USA) containing 3% penicillin/streptomycin to isolate the ECs. Endothelial growth medium (EGM‐2; Lonza, USA) containing collagenase type IV (0.1%, 1 mg/mL; Gibco, USA) was injected into the umbilical vein followed by 20 min incubation at 37°C. Following the incubation, the suspended cells were centrifuged at 1297 ×g for 10 min and cultured with the EGM‐2 medium.

### 2.6 Reconstituting kidney‐like three dimensional structure

MLCs were co‐cultivated with ELCs with total numbers of 6 × 10^4^ and 3 × 10^5^ cells in 10:5 ratio, respectively (this group was hereafter named ME). In another experiment, we combined MLCs, ELCs and HUVECs at the total number of 4 × 10^5^ cells with 10:5:2 cell ratio (this group was named MEH). These aggregates were incubated with NPC self‐renewal (NPSR) medium,^25^ in absence of heparin and BMP7 for 10 days in vitro. NPSR medium was composed of DMEM/F12 (Invitrogen, USA), supplemented with 10% FBS, 20 ng/mL EGF, 2% B‐27 supplement (Gibco, USA), 2 mmol/L L‐glutamine (Gibco, USA), 0.1 mmol/L β‐mercaptoethanol (Sigma‐Aldrich, USA), 10 μM Rho‐associated protein kinase (ROCK) inhibitor (Sigma‐Aldrich), 200 ng/mL basic fibroblast growth factor (BFGF; Royan Biotech, Iran), 10 ng/mL mouse leukaemia inhibitory factor (LIF; Royan Institute, Iran) and 1 μM CHIR99021 (Stemgent, USA). We used quantitative reverse transcription PCR (qRT‐PCR) analysis for kidney‐specific markers to identify ideal cell ratios forming these groups. The samples were obtained from three to five independent biological replicates, and all reactions were performed technically duplicates.

### 2.7 Grafted kidney micro‐tissue formation by the chick embryo chorioallantoic membrane (CAM) assay

The generated kidney micro‐tissue was transplanted onto chorioallantoic membrane (CAM). Fertilized eggs, from Hy‐Line W‐36 laying hens, were supplied from a commercial farm (Iran). The eggs were kept horizontally at 37°C and incubated in 60% humidity condition. The eggs were firstly cracked under a sterile biological laminar‐flow hood, and the yolk‐containing embryo was then moved into a larger sterile petri‐dish. The first surrogate shell, with just marginally (3–4 g) heavier than the eggshell, was then utilized to transfer the yolk‐embedded embryo. The shell was then covered with plastic wrap and incubated for 60 h with forced air circulation at 37°C and 60% humidity. This period of time was defined as embryonic day 0 (ED0). On ED2.5, a recipient surrogate shell, containing the yolk‐embedded embryo, was transferred again. It was subsequently sealed with regular plastic tape and cultured for the additional 5 days. The renal micro‐tissues were put on the intact CAM at ED7.5, and the embryo was maintained in the incubator for seven additional days. The generated renal micro‐tissues were consequently picked up for further evaluations.

### 2.8 Flow‐cytometry analysis

The flow‐cytometry was performed to quantify MLCs at 4 × 10^6^ cells, based on the previously published study.^25^ Briefly, the cells were dissolved as single cells, using trypsin/EDTA for 5 min at 37°C and washed with PBS. After centrifugation at 981 ×g for 5 min, the cell pellet was fixed in 2% paraformaldehyde for 15 min at 4°C. The samples became penetrable and blocked. The cells were next incubated with primary antibody solution for overnight, followed by washing twice with 1% BSA in PBS. Subsequently, they were treated with a secondary antibody for 2 h at room temperature. Quantification was performed using a flow‐cytometer (FACS Calibur; BD Biosciences, USA) and flowing software, version 2.5.1 (RRID: SCR_015781). All experiments were repeated three times. List of the primary and secondary antibodies, utilized in this study, are provided in Table 1.

**TABLE 1:** List of antibodies used for immunostaining and flow‐cytometry.

Antibody	Source	Catalogue no.	Host	Concentration
Cytokeratin 18	Chemicon	cb177	Mouse	1:200
E‐cadherin	Santa Cruz	Sc‐8426	Mouse	1:200
Anti‐mouse Alexaflour488	1nvitrogen	A‐11001	Goat	1:1000
Anti‐mouse Alexaflour594	Invitrogen	A‐11020	Goat	1:500
E‐cadherin (24E10)	Cell signalling	#3195	Rabbit	1:100
Vimentin (D21H3)	Cell signalling	#5741	Rabbit	1:100
Cd34	Bioss‐BS	2038‐R	Rabbit	1:200
Anti (rabbit) Alexa Fluor 488	Invitrogen	A11034	Goat	1:500
Aquaporin 1	Biorbyt Biocompare.com	orb10122	Rabbit	1:200
Anti (rabbit)	Biorbyt	orb688925	Goat	1:500
Wt1	R&D systems	AF5729	Goat	1:200
Anti (rabbit) Alexa Fluor 488	Invitrogen	A21206	Donkey	1:500

### 2.9 Histological studies

In this experiment, the cells and micro‐tissues were respectively fixed 20 min and overnight in 4% paraformaldehyde (PFA; Sigma‐Aldrich, USA) at room temperature. To analyse micro‐tissues histologically, using immunoflourescence assay, they were initially embedded in paraffin and sectioned at 5 μm thickness using microtome (Thermo Fisher Scientific, USA). After deparaffinization and rehydrating the sections on the slides, they underwent an antigen retrieval phase using retrieval solution, followed by permeabilization in PBS, containing 0.5% Triton X‐100, for 5–20 min at room temperature. The samples were next rinsed twice with DPBS and fixed in 4% paraformaldehyde for 20 min. The fixed cells were then rinsed three times in DPBS before incubating in a blocking solution (composed of 0.1% Triton X‐100, 10% secondary antibody host serum and 0.5% BSA) for 1 h at room temperature. Subsequently, the primary antibody was added to the cells and they were incubated at 4°C overnight or 2 h at room temperature (Table 1). The cells were then washed three times in PBS and incubated with the appropriate secondary antibodies (Table 1) for 1 h at 37°C. Cell nuclei were counterstained with 4´,6‐diamidino‐2‐phenylindole (DAPI; Sigma‐Aldrich, USA) for 1 min. The immunofluorescent stained cells were visualized under a microscope (IX71; Olympus, Japan) and captured by an Olympus DP72 digital camera. Images of immunofluorescence cells were quantified using ImageJ (RRID: SCR‐003070) program. In this study, a negative sample was generated for each staining and the exposure time was reduced to less than 500, in order to prevent fluorescence intensity. In terms of immmunohistochemically staining, 5 μm paraffin‐embedded micro‐tissue sections were initially blocked by exposing to 3% H_2_O_2_ for 10 min. It was next followed by adding antigen retrieval through incubation in retrieval solution for 1 h. After cooling it at room temperature, non‐specific sites were blocked. Incubation with the primary antibody was performed in a moist chamber at 4°C. The nuclei were counterstained with haematoxylin. Table 1 lists the antibodies. After deparaffinization and hydration, Masson's trichrome (MT) staining (Sigma‐Aldrich, USA) was carried out on some sample slides (Figure S1). After fixation and paraffin embedding in four groups, 5 mm thickness sections were prepared using a microtome and they were stained with haematoxylin and eosin.

### 2.10 Quantitative reverse transcription PCR

RNeasy Mini Kit (Qiagen, Germany) was used to extract total RNA in accordance with the manufacturer's instructions, followed by converting 1 mg of the total RNA into cDNA (Thermo Fisher Scientific, USA). qRT‐PCR was performed by StepOnePlus Real‐Time PCR system (Applied Biosystems, USA) from cDNAs, using SYBR Green reagent (Takara Bio, Japan), according to the previously published protocols.^26^ Findings were normalized to glyceraldehyde 3‐phosphate dehydrogenase (*GAPDH*) expression. Expression level of different genes was calculated using 2−^∆∆Ct^ method. The samples were obtained from three independent biological replicates and all reactions were performed in duplicate. OLIGO Primer Analysis Software is used to design and analyse PCR primers. Table 2 shows list of the utilized primers in this research.

**TABLE 2:** List of primers used for qRT‐PCR.

Gene	Forward primer sequence	Reverse primer sequence
*Aqp1*	5′‐AGGCTTCAATTACCCACTGGA‐3′	5′‐GTGAGCACCGCTGATGTGA‐3′
*Aqp2*	5′‐ATGTGGGAACTCCGGTCCATA‐3′	5′‐ACGGCAATCTGGAGCACAG‐3′
*Cd133*	5′‐AGAAAGTGAAGAAGATCCTTGCC‐3′	5′‐GAACCAGAACAAATTCAGAGGG‐3′
*Gapdh*	5′‐CAACTC CCA CTC TTC CAC TT‐3′	5′‐GCA GCG AAC TTT ATT GAT GGT A‐3′
*Gata3*	5′‐CTCGGCCATTCGTACATGGAA‐3′	5′‐GGATACCTCTGCACCGTAGC‐3′
*Nphs1*	5′‐ACATGGCCTTCCCCGGACAC‐3′	5′‐AAAGGGCAGAGAACCAGGCTC‐3′
*Podxl*	5′‐CAGCCTGCCGCTCATCATCAC‐3′	5′‐TCTGTGAGCCGTTGCTGGTCC‐3′
*Wt1*	5′‐AGCACGGTCACTTTCGACG −3′	5′‐GTTTGAAGGAATGGTTGGGGAA‐3′
*Umod*	5′‐GACCTGGATGCTGCTGGTAAT‐3′	5′‐GTGGCGTTGTTGTGGCATTC‐3′
*E‐Cadherin*	5′‐TCGGAAGACTCCCGATTCAAA‐3′	5′‐CGGACGAGGAAACTGGTCTC‐3′
*Cd105*	5′‐CTCAGGGCCCGTCCACACCA‐3′	5′‐AGGGGCGTGGGTGAAGGTCA‐3′
*Cd45*	5′‐ACCACCAGGTGAATGTCAATTT‐3′	5′‐CTTGCTTTCCCTCGGTTCTTT‐3′
*Foxd1*	5′‐CTTCTCCATCGAGAGCCTCAT‐3′	5′‐CTGTCCCTTGGTGCAGAGTC‐3′
*Cd34*	5′‐GGTAGCTCTCTGCCTGATGAG‐3′	5′‐TGGTAGGAACTGATGGGGATATT‐3′
*Scf*	5′‐AGACACAAGTGAGTAGGGCAC‐3′	5′‐TCCCGGAGCGATTTTCTTGG‐3′
*Lrg4*	5′‐CCGCTGCCTGCTTGCCTGAA‐3′	5′‐TCCTGGTGACACGCCGCTTC‐3′
*Pax8*	5′‐CAGAAGGCGTTTGTGACAATGA‐3′	5′‐TGCACTTTGGTCCGGATGAT‐3′
*Cd106*	5′‐GGTGGCTGCACAGGTTGGGG‐3′	5′‐ACCCACAGGGCTCAGCGTCA‐3′
*Cd24*	5′‐TTCTGGCACTGCTCCTACC‐3′	5′‐GCGTTACTTGGATTTGGGGAA‐3′
*Synpo: Synaptopodin*	5′‐CCTGAACCTACCAAGCAGCA‐3′	5′‐AGACAGTGCGTGATGGAGTG‐3′
*Nphs2* (*podocin*)	5′‐CTGCCAGCTGGGCTTCAGCA‐3′	5′‐CTGGGGTGCCCGACAGAATC‐3′
*C kit* (*Cd117*)	5′‐CTAAAG ATG AAC CCT CAG CCT‐3′	5′‐GCA TAA CAC ATG AAC ACT CCA‐3′
*Sox9*	5′‐CCA GCG TTT AAC CTT CAAGAC‐3′	5′‐ATT TAA CAA CAG ATG ACC ATA CCC‐3′
*Cited1*	5′‐AAGGCACAGCACCCACTCGG‐3′	5′‐GGGTAGTGCAGGACAGTCAC‐3′
*Cd44*	5′‐TAGATCCCTCCGTTTCATCCA‐3′	5′‐GGTTACATTCAAATCGATCTGCTG‐3′
*Ck18*	5′‐ATT TCA GTC TCA ACG ATG CC‐3′	5′‐GAA CTC TGG TGT CAT TAG TCT C‐3′
*Aldh1a2*	5′‐AGGTGGATATAGACAAGGCAGT‐3′	5′‐CCGCCATTTAGGGATTCCATAG‐3′
*Cd90*	5′‐CTC TCC TGC TCT CAG TCTTG‐3′	5′‐AGT TAT CCT TGG TGT TAT TCT CAT‐3′

### 2.11 Statistical analysis

Mean and standard deviation of at least three biological and two technical replicates were used to represent all data. In Graph Pad Prism software (version 8, USA), statistical analysis was performed using the unpaired, two‐tailed Student's *t‐test* for pairwise comparisons or one‐ and two‐way analyses of variance, followed by the proper post hoc test for assessment of differences between more than two groups. A *p*‐value of <0.05 was considered statistically significant. ANOVA (one‐way and two‐way) analysis of variance was used to determine significant differences, among the groups, with Tukey's post hoc test.

## 3 RESULTS

### 3.1 Characterization of epithelial‐like cells

**FIGURE 2:**
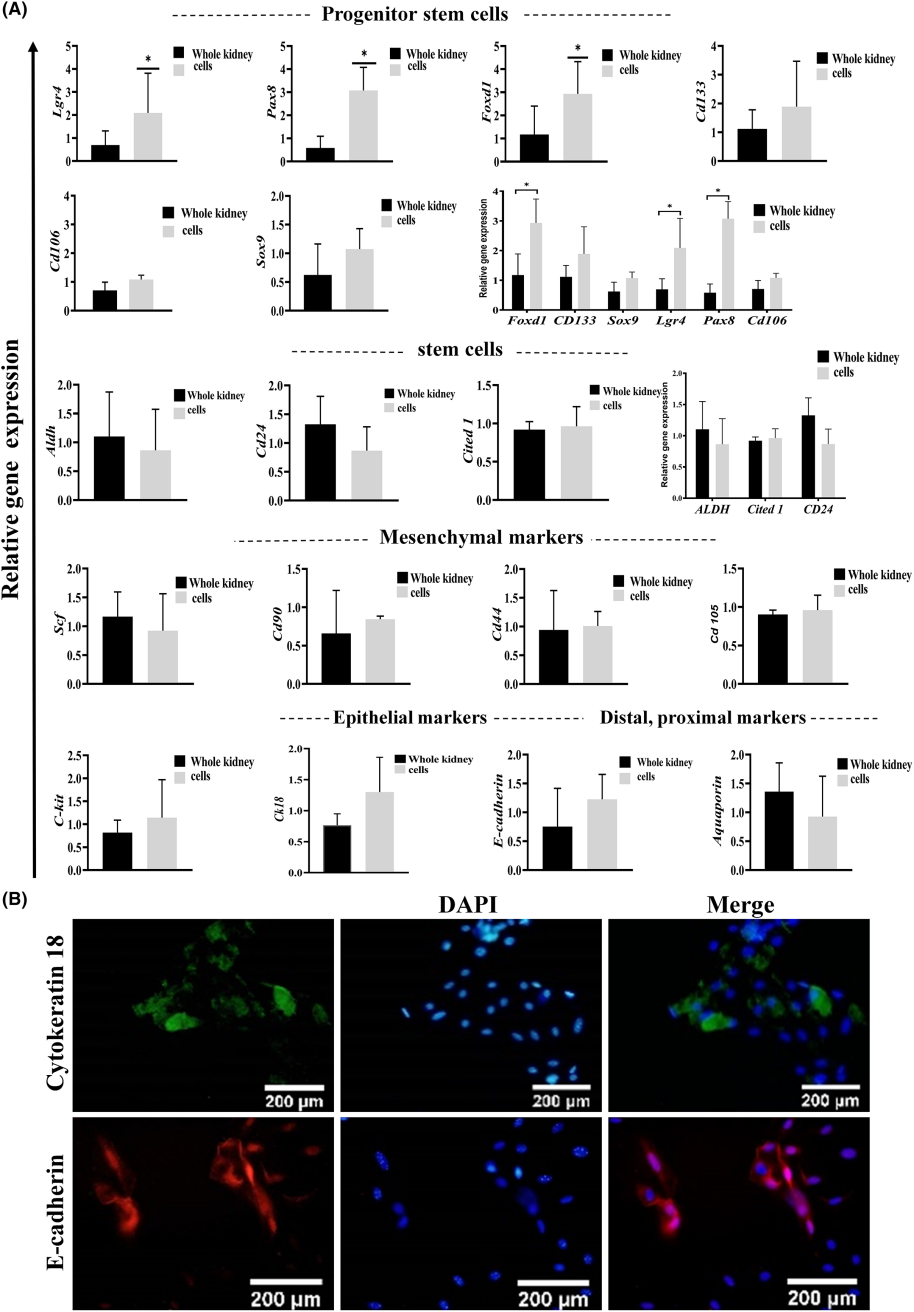
ELCs characterization. (A) qRT‐PCR analysis at 3 × 10^6^ cells in three independent biological replicates, and technically duplicates for progenitor stem cell markers (*Cd133*, *Sox9*, *Lgr4*, *Pax8* and *Cd106*), stem cell markers (*Aldh*, *Cited1* and *Cd24*), mesenchymal markers (*Scf*, *C‐kit*, *Cd90*, *Cd44* and *Cd105*), epithelial markers (*Cytokeratin* and *E‐cadherin*), distal and proximal tubule markers (*Cdh1* and *Aquaporin1*). (B) Immunocytochemistry staining about 100,000 cells in each well of the 4‐well for epithelial markers (E‐cadherin and cytokeratin18) expression.

Colony formation was visible since the second day of culture and it reached the maximum size on Days 7–8. In some of the colonies, up to 40000 cells were even detected. These cells started mimicking the mesenchymal fate after 8 days. ELCs displayed predominantly cobblestone morphology, as typical characteristic features of epithelial cells (Figure 1A). qRT‐PCR was performed technically duplicate and biologically three times (about 3 × 10^6^ cells). Analysis revealed expression of *Ck18*, *Cdh1* (epithelial tubular markers), *Cd133*, *Cd106*, *Lgr4*, *Sox9*, *Pax8*, *Foxd1*, *Aldh*, *Cited1*, *Cd24*, *Scf*, *C‐kit* (stem/progenitor cell markers) and *Aqp1* (proximal tubular marker) in ELCs (Figure 2A). On the other hand, *Cd105*, *Cd90* and *Cd44* (mesenchymal markers) expression levels were not significantly increased, or expressed less than the average expression levels in the whole organ (Figure 2A). Furthermore, immunofluorescent staining method of about 100000 cells in each well of the 4‐well showed expression of the two epithelial cell‐specific markers, namely Cytokeratin 18 and E‐cadherin (Figure 2B). A heterogeneous cell mixture of proximal aqp1^+^ and distal cdh1^+^ gave rise from the previously indicated method of isolation.^4^ In this protocol ELCs were isolated, followed by sub‐culturing the cells for three passages. Analysis of the cells indicated typically epithelial characteristics, while they expressed tubular markers. To perform further studies, the cells were cultured on collagen gel in order to expand the monolayer cells. Bright‐field images of ELCs (passages 1‐3) demonstrated a cuboidal‐like morphology (Fig. 1.D). In heterogeneous populations at 2 × 10^6^ cells expression levels of E‐cadherin and Vimentin antibodies were analyzed by flow‐cytometer on days 4 and 7 (Fig. 1.F). Immunofluorescent staining of ELCs determined E‐cadherin and cytokeratin expressions (Figure 2B). Crystal violet staining or clonogenic assay results were visible in Figure 1C, following the culture of 1 × 10^5^ kidney tissue‐derived cells, about 150 colonies within the average dimension of 1.21 ± 0.37 mm^2^ were detected. Analysis of the triplicate repeat of ELCs showed self‐organizing capability of these cells in the third passage, while the total cell number was 4 × 10^5^ in each of 4‐well plate. This demonstrated podocyte and whole kidney markers (Figure S1).

### 3.2 Characterization and multi‐lineage differentiation of mesenchymal‐like cells

**FIGURE 3:**
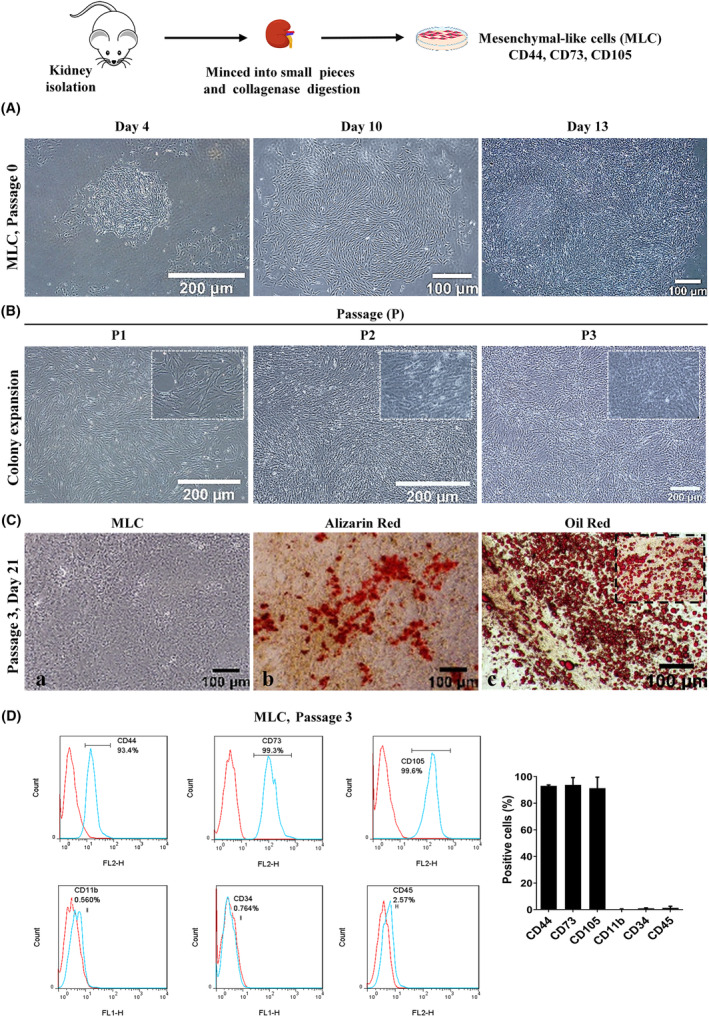
Mesenchymal like cells (MLCs) pick up and characterization. (A) MLCs, passage 0 (Days 4, 10 and 13). (B) MLCs pick up and expansion on Matrigel, passages 1–3. (C) Osteogenic and adipogenic differentiations of about 300000 MLCs cells in each of 6 well in three independent biological replicates and technically duplicates, passage 3: (a) osteogenic differentiation cells, Day 21, (b) alizarin red staining (osteogenic differentiation cells), (c) oil red staining (adipogenic differentiation cells). (D) Flow‐cytometry at 4 × 10^6^ cell number for mesenchymal markers (Cd105, Cd73 and Cd44) as well as endothelial markers (Cd11b, Cd45 and Cd34) in MLC, passage 3.

MLCs displayed predominantly spindle‐shaped (a classical MSC‐like phenotype) morphology. Figure 3.A shows morphology of the cells, during Days 4–13 of the culture. Analysis of the bright‐field images of MLCs (passages 1–3) demonstrated a dynamic fibroblast‐like and spindle‐shaped morphology (Figure 3B). These cells were cultured up to 11 passages without determining any evidence of senescence. Although the kidney cells were unable to differentiate into bone, adipose or cartilage in normal organogenesis, MLCs (about 300000 cells in each of 6 well) was performed technically duplicate and biologically tree times, like the other sources of MSCs had an ability to convert into all of these tree lineages. In this regards, analysis of the presence of calcium, using Alizarin Red staining, as well as the lipid droplets, using Oil Red staining, in the culture medium demonstrated MSC role of MLCs (Figure 3C). The earliest mineralized aggregates were formed 1 week after starting differentiation in some areas of the cell culture. It was subsequently increased during cultivation. Three weeks later, starting bone differentiation was demonstrated by detecting red colour in some place of the culture medium, due to the staining with Alizarin Red. MLCs were also assessed for adipogenic differentiation. On the fifth day of differentiation, the first lipid droplets appeared in some cells. This was gradually spread and included many cells by the end of the culture period. Red colour was positively detected in these droplets, using Oil Red staining (Figure 3C). To detect mesenchymal properties, flow‐cytometry was utilized in 4 × 10^6^ cells number. Results showed that MLCs highly expressed special mesenchymal surface markers, including Cd44 (93.4%), Cd73 (99.3%) and Cd105 (99.6%), while these cells did not express haematopoietic (Cd11b, Cd34 and Cd45) markers (Figure 3D). This data suggested that numerous cells expressed typical mesenchymal markers. To perform further studies, MLCs were cultured on monolayer cells in order to study phenotypic characteristics. Overall, our data showed that MLCs had many typical characteristics of MSC.

### 3.3 Generation and evaluation of in vitro kidney micro‐tissues

**FIGURE 4:**
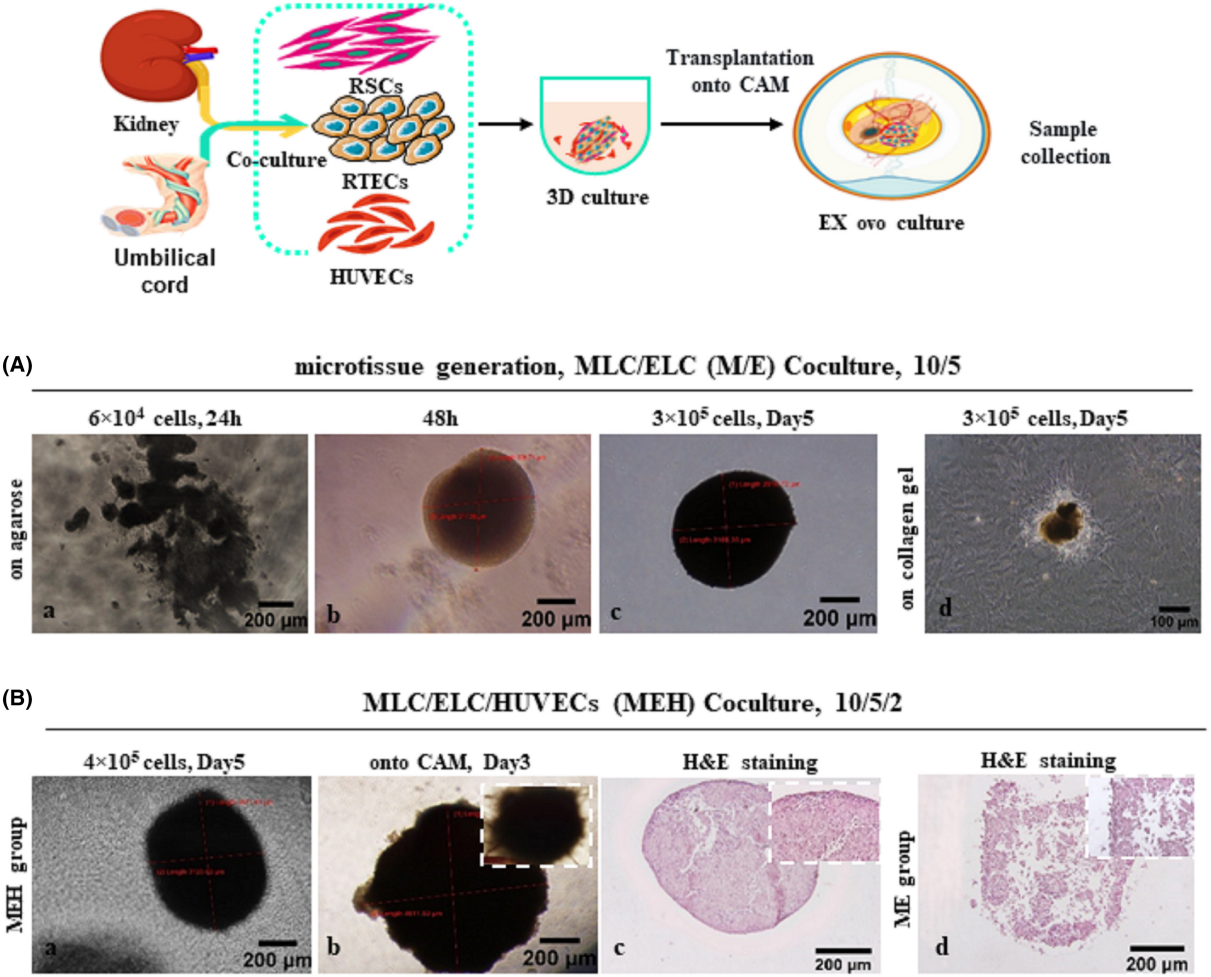
Micro‐tissue generation. (A) MLC/ELC co‐culture (ME group ratio: 10/5): (a, b, c) ME group on agarose, (a) 24 h, 6 × 10^4^ cells, (b) 48 hours, size: 350 μm, (c) Day 5, 3 × 10^5^ cells, size:2‐4 mm, (d) Day 5, 3×10^5^ cells on collagen gel. (B) MLC, ELC and human umbilical vein endothelial cells (HUVECs) co‐culture (MEH group): (a) ratio: 10/5/2, 4 × 10^5^ cells, (b) ratio: 10/5/2, in 96‐well U‐bottom ULA plates, size: 3–4 mm, (c) Ex ovo chorioallantoic membrane or CAM explanting of MEH (CE MEH), Day 3, (d) Haematoxylin and eosin staining of CE MEH and ME groups.

**FIGURE 5:**
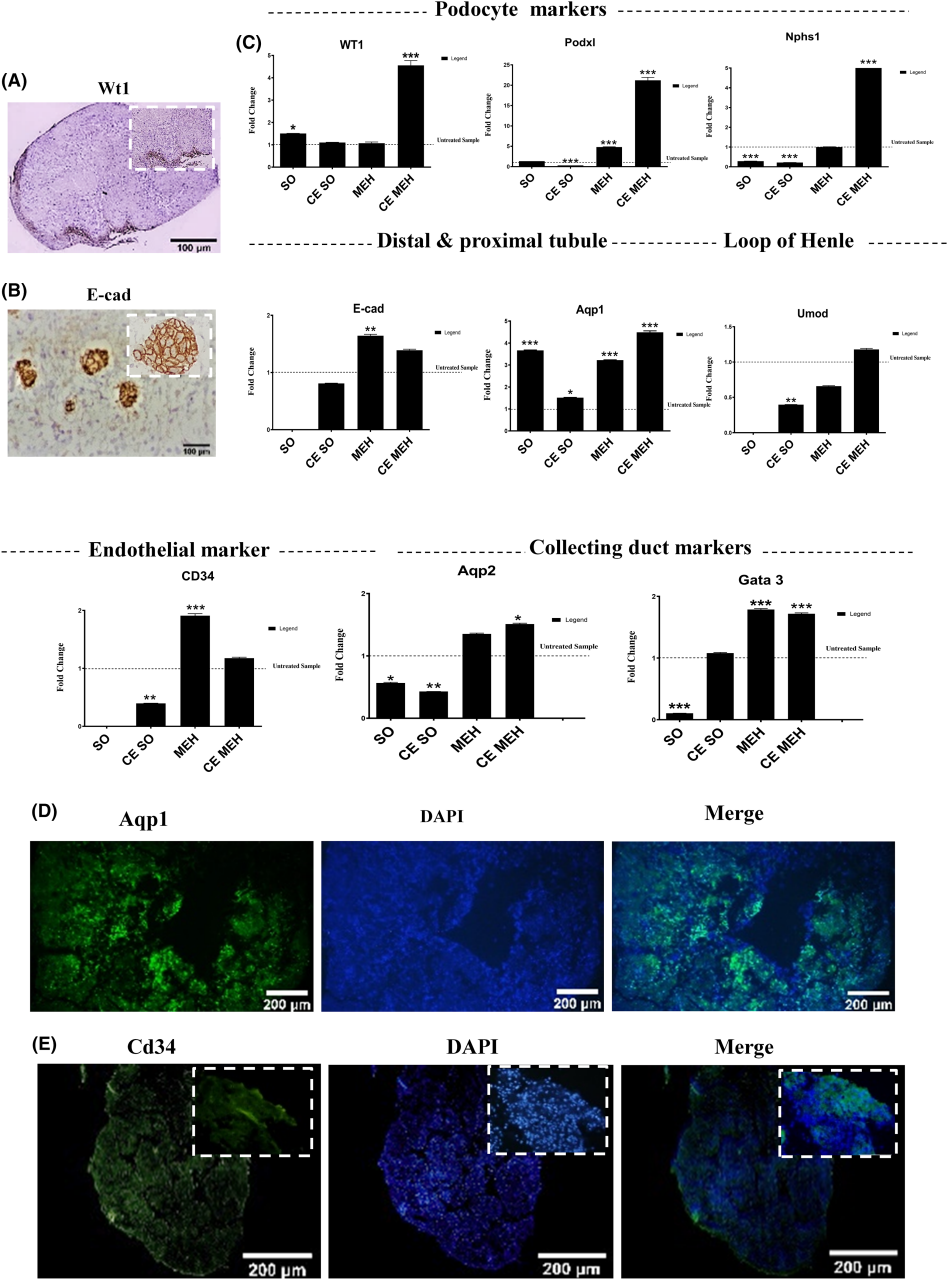
Microtissue evaluation. (A, B) Immunohistochemistry in CE MEH group, for Wt1 and E‐cad expression. (C) qRT‐PCR analysis for renal specific genes in four groups (self‐ organized, CAM explants self‐organized, MEH and CE MEH groups), podocyte markers (*Nphs1*, *Podxl* and *Wt1*), distal and proximal tubule markers (*Cdh1*, *Aqp1*, *loop of Henle marker*, *Umod*), endothelial markers (*Cd34*, collecting duct markers, *Aqp2* and *Gata3*), *n* = 3 (D, E) Immunofluorescent staining of CE MEH group for the proximal tubule markers (Aqp1, endothelial marker, CD34), *n* = 3.

Dense multicellular aggregates of mesenchymal‐epithelial (ME) were created 48 h after the co‐culture (Figure 4A). qRT‐PCR analysis showed that the best ratio of micro‐tissue formation was 10:5:2 MLC:ELC:HUVECs at 4 × 10^5^ cells number. Figure 4A showed the microscopic fate of ME micro‐tissue generation on agarose: (a, b) at 6 × 10^4^, (c) 3 × 10^5^ cells number and (d) collagen gel at 3 × 10^5^ cells number. Bright‐field haematoxylin and eosin staining of the ME and MEH groups are shown in Figure 4B. These result demonstrated low condensity in the ME group compared to the other groups. qRT‐PCR results in three independent biological replicates and two technical repeats showed that expression levels of *Cdh1* (epithelial marker), *Gata3* (distal markers), *Aquaporin1* (proximal marker), *Cd34* (endothelial marker), *Wt1*, *Nphs1*, *Podx1* (podocytes markers) were significantly higher in MEH compared to ME. These expression levels were further increased after CAM transplantation (Figure 5C). Protein expressions of E‐cad, Aqp1, Cd34 and Wt1 were confirmed using immunohistochemistry and immunohistofluorescent techniques (Figure 5A, B, D, E).

### 3.4 Ex vivo transplantation of the self‐ aggregated MLCs and ELCs with HUVECs

The previous studies determined that mesenchymal and endothelial cell incorporations to kidney structures promoted cell development and maturation.^24^ We hypothesized that transplantation of MEH group into the CAM would increase maturation efficiency. To determine optimal self‐condensation combination, different ratios of MLCs:ELCs:HUVECs (10:5:5, 10:6:3 and 10:5:2) were examined in three organoids and three replicates. Findings showed that 10:5:2 ratio of this combination formed the best self‐condensation. Thus, this ratio was chosen for further studies (Figure S2). To generate a three dimensional (3D) transplantable condensed structure, these heterogeneous cells were cultured for 7–10 days (Figure 4A) based on the previously reported protocol.^27^ It was subsequently transplanted into 7 days old CAM at three times. Ten days after transplantation, the micro‐tissue generated a condensed form. Findings obtained from the haematoxylin and eosin staining showed lower tissue density in the ME groups in comparison with the other groups. This could confirm the potential effect of HUVECs on improving density of micro‐tissues (Figure 4B). Analysis of MEHRMs in three times revealed increased expression of renal signature genes, including *Aqp1* (proximal tubule), *Cdh1* (distal tubule), *Umod* (loop of Henle), *Wt1*, *Podxl* and *Nphs1* (podocyte markers).

## 4 DISCUSSION

Micro‐tissues aggregated from renal clonogenic cells are 3D constructions mimicking the major characteristics^28^ of the original kidney tissue. They are appropriate platforms to understand potential mechanisms beyond the adult kidney physiology and disease condition.^12^ In this regard, Schutgens and colleagues separated primary renal tubular cells (RTECs) from renal cortical tissue. Six days after plating in Matrigel, renal tubuloids, including simple cuboidal epithelial cells from proximal tubule, loop of Henle, distal tubule and collecting duct segments, were generated and sustained for as many as 20 passages.^12^ In this approach 3D kidney structures was generated by using the culture system based on Matrigel.^12^ Nevertheless, utilizing Matrigel bears several disadvantages, including pathogen transmission,^24^ inter‐batch variability and technical challenges in handling. Matrigel is derived from mouse tumour sarcoma and the exact composition of this xenobiotic product is not well‐defined.^29^ In addition, the previously indicated study could not detect any endothelial or stromal component in the tubuloids.^12^ To solve this problem, protective co‐culture systems, containing endothelial and stromal cells provided appropriate physiological niche and promoted organoid self‐organization^28^ and maturation.^11^, ^30^ Studies determined a variety of paracrine agents secreting from mesenchymal cells and they were capable to promote 3D kidney structures maturation.^24^, ^31^, ^32^ In this regard, ECs played vital roles in transferring oxygen and nutritional materials as well as secreting several angiogenic growth factors. Several studies established heterotypic cellular aggregates to create high‐quality and vascularized 3D kidney models.^24^, ^33^ Khoshdel‐Rad et al. differentiated hPSCs into nephrogenic intermediate mesoderm (IM) stage. These IM cells were then co‐cultured with HUVECs and BM‐MSCs at a ratio of 10:7:2, respectively. After an 11‐day co‐culture period, the culture revealed 3D human renal microtissues expressing markers indicative of both glomerular and renal tubular structures. Consequently, the integration of endothelial and mesenchymal cells notably contributed to the maturation of the renal microtissues.^24^ Generating kidney organoids from human hPSCs presents numerous unresolved obstacles. These hurdles encompass immature renal cells, an incipient vascular system and the potential risk of tumorigenesis.^11^ In another study, Yang et al. introduced hybrid 3D spheroids by co‐culturing immortalized podocytes, human MSCs, and HUVECs at a 1:1:1 ratio, successfully recapitulating the native glomerulus‐like condition. Subsequent transplantation of these hybrid cell spheroids into the renal cortex resulted in enhanced engraftment efficiency and improved kidney function.^34^ Immortalized human cell lines do not exhibit the histological characteristics typical of kidney cells. Additionally, the process of immortalization disrupts the accurate assessment of the cell cycle and its association with various diseases.^24^, ^35^ In the current study, first we presented an approach to promote sufficient number of the isolated ELCs and MLCs, followed by developing a 3D organotypic co‐culture model by using HUVECs. Thus, the latter cells supported further differentiation of renal cells. MEHRMs were grown in the ULA 96‐well round‐bottom plates that are ideal for high identity performance. Under supporting 3D co‐culture situation, seeded mixed cells were proliferated, followed by condensing and differentiation into the renal lineage. For this purpose, MEHRMs, especially those which were transplanted on CAM, revealed increased expression of renal gene markers, such as *Aqp1* (proximal tubule), *Cdh1* (distal tubule), *Umod* (loop of Henle), *Wt1*, *Podxl* and *Nphs1* (podocytes marker), *Gata3*, *Aqp2* (collecting duct markers) and endothelial marker (*CD34*). This data confirms findings from two other studies on the transplantation of kidney organoids onto CAM, indicating an enhancement in the expression of kidney‐specific genes.^24^, ^36^ The renal clonogenic cells generated micro‐tissue are 3D assemblies, mimiking the major characteristics of normal kidney tissue. Subsequent implantation of these aggregates into the CAM further promoted maturation of micro‐tissues. CAM is an incomplete immune system in host which can preserve viability of small tissues without any species‐to‐species reaction.^37^, ^38^ Soft substrates with mechanical characteristics similar to the primitive fetal milieu, like CAM, showed to improvement of maturation of renal microstructures in different studies.^33^, ^36^ Evidences showed that expression levels of renal‐specific markers were increased in the grafted group.^24^ Paracrine factors produced by ECs, played important role in renal organogenesis and regeneration.^3^, ^5^, ^39^ In the present study, MEH groups, especially CAM explanted MEH (CE MEH), showed a significant increase in the expression level of the renal‐specific genes, such as *Aqp1* (proximal tube marker), *Wt1*, *Podx1*, *Nphs1* (podocyte markers), *Cdh1*, *Gata3* (distal tube maker) and *Cd34* (endothelial marker) compared to the other groups (Figure 5C). The previous researches showed that stromal cells produced cytokines and growth factors, while they are important for kidney development and regeneration.^3^, ^5^ Additionally, several ECM has been shown to support and associate with epithelial and stem cell behaviours.^37^ Collagen gels, due to the focal kinase pathway (FAK), played a fundamental role in epithelial morphogenesis^40^ through conduction of mechanical stimuli.^40^, ^41^, ^42^ Therefore, we cultured and expanded ELCs on collagen (Figure 1B). In this study, we also used Matrigel ECM for MLCs expansion (Figure 3B). Generation of mini‐guts from mouse intestinal stem cells within a floating collagen gel was performed.^43^ Collagen gel has a critical role in epithelial tubulogenesis and branching in a spatiotemporal manner.^44^ In this regards, we studied how micro‐tissue was self‐organized into macroscopic tubes, using collagen gel contraction, agarose and ULA 96 wells (Figure 4A, B). Evidences demonstrated the physical manipulation potential of collagen properties. This could affect shape of the embedded cells.^45^ But, in our study, micro‐tissue was not formed well with collagen substrate. In contrast, low attached plate and agarose demonstrated better output. This approach is planned for studies focusing on many types of nephron segment and collecting duct cells. This is also used for maintenance, as a frame to recapitulate more complex experiments to determine epithelial and mesenchymal cell pathology‐associated kidney diseases. However, these micro‐tissues provided a rapid cost‐effective protocol for producing in vitro models, upon obtaining them by tissue biopsy. Despite demonstrating usefulness, the current method faces some drawbacks. As a case, clonogenic cells phenotype may be changed in the culture after a limited number of passaging. Furthermore, our observations indicate that the 10:5:2 ratio of this combination resulted in the most favourable self‐condensation outcome. However, it's important to acknowledge that these findings don't completely explain the functional properties within the condensate. Further exploration into the underlying mechanisms and the impact of other factors is necessary to fully grasp this process. Thus, our conclusions about the optimal ratio should be approached with caution, and more research is required to validate and improve these findings.

## 5 CONCLUSION

In conclusion, our study addresses several critical aspects, including the economic feasibility, and, novelty in generating 3D renal micro‐tissues. Our streamlined method significantly reduces costs by minimizing the use of expensive materials, such as growth factors and growth factor‐reduced Matrigel, thereby enhancing the economic viability of organoid production. Furthermore, while similar co‐culture systems exist in the literature, our approach distinguishes itself through the unique combination of kidney‐specific mesenchymal and epithelial cells. This included various parts of the nephron, collecting duct, stromal and ECs. Supportive cell attendance provided a micro‐physiological niche that recapitulated in vitro renal tissue. This distinct composition is pivotal in generating 3D renal micro‐tissues and represents a novel contribution to the field. Additionally, our method demonstrated efficiency in yielding a substantial number of cells, maintained over numerous passages without senescence. This novel procedure could help generate low price and fast configuration of in vitro renal models which may potentially be used in patient‐derived disease modelling. While our approach shows promise in bridging the gap between organoid models and in vivo systems, it's important to acknowledge the inherent limitations. One significant challenge is the lack of reproducibility in organoid generation. Despite efforts to standardize protocols and ensure statistical reproducibility across multiple biological replicates, variability between organoids derived from different individuals and intra‐organoid heterogeneity remain significant challenges. Addressing these factors is pivotal for advancing the reliability and translational potential of organoid research. Future investigations should prioritize strategies aimed at mitigating such variability, thus fostering more robust and reproducible experimental outcomes in the realm of organoid biology.

## AUTHOR CONTRIBUTIONS

**Fatemeh Abdollahzadeh:** Formal analysis (equal); writing – original draft (equal). **Niloofar Khoshdel‐Rad:** Writing – review and editing (supporting). **Khadijeh Bahrehbar:** Methodology (supporting). **Saiedeh Erfanian:** Methodology (supporting). **Vahid Ezzatizadeh:** Writing – review and editing (supporting). **Mehdi Totonchi:** Supervision (lead). **Reza Moghadasali:** Supervision (lead).

## CONFLICT OF INTEREST STATEMENT

The authors declare no conflicts of interest.

## Supporting information


Figure S1.

Figure S2.

## Data Availability

The authors confirm that the data supporting the findings of this study are available within the article and its supplementary materials.
