# Supplementary figures and images for: Enhancing maturity in 3D kidney micro‐tissues through clonogenic cell combinations and endothelial integration
Source: J Cell Mol Med. 2024 May 31;28(11):e18453. doi: 10.1111/jcmm.18453 (PMC11140233; doi:10.1111/jcmm.18453)

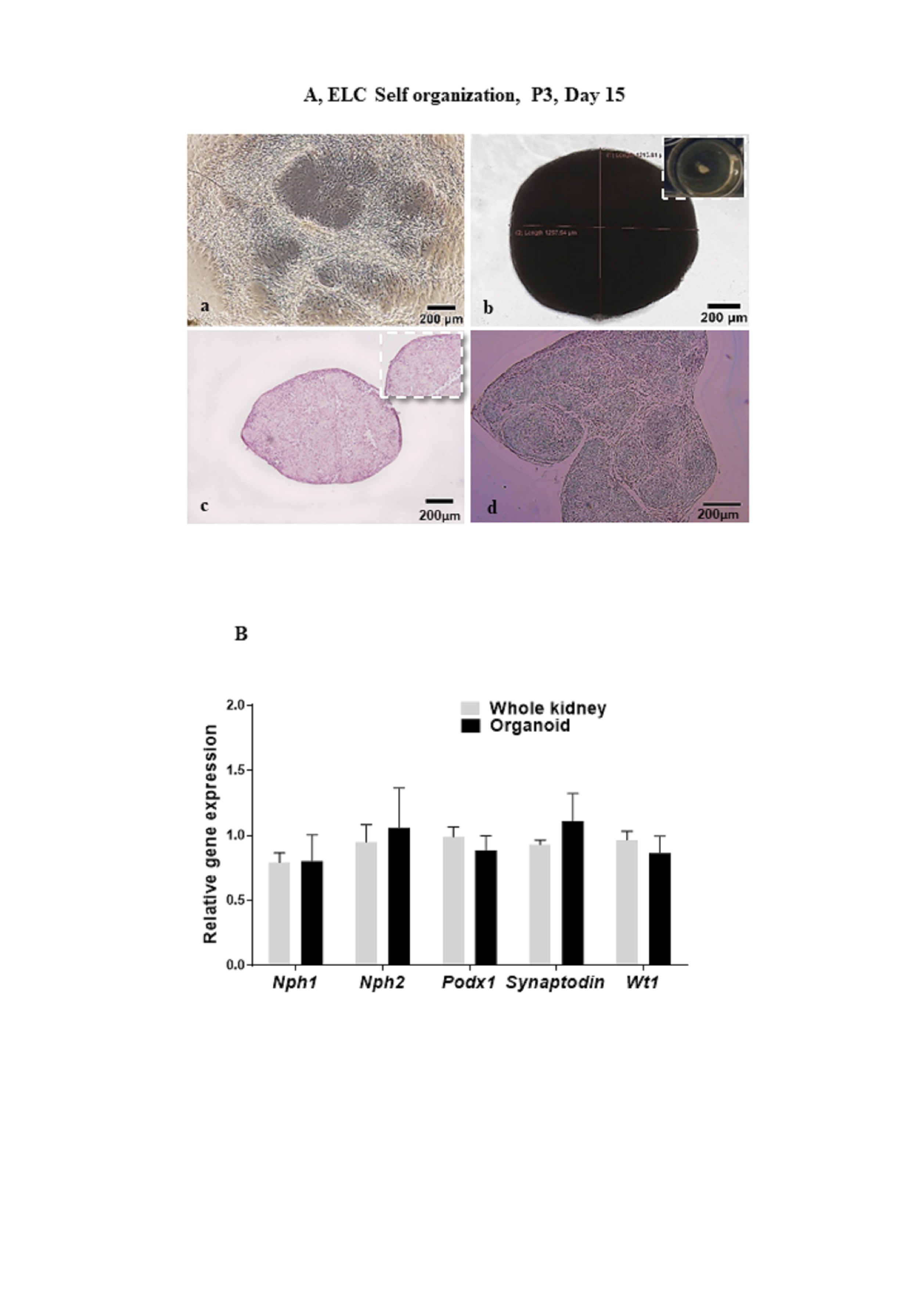

Supplement: Supplementary file 1 — Figure S1. [file JCMM-28-e18453-s001.jpg]

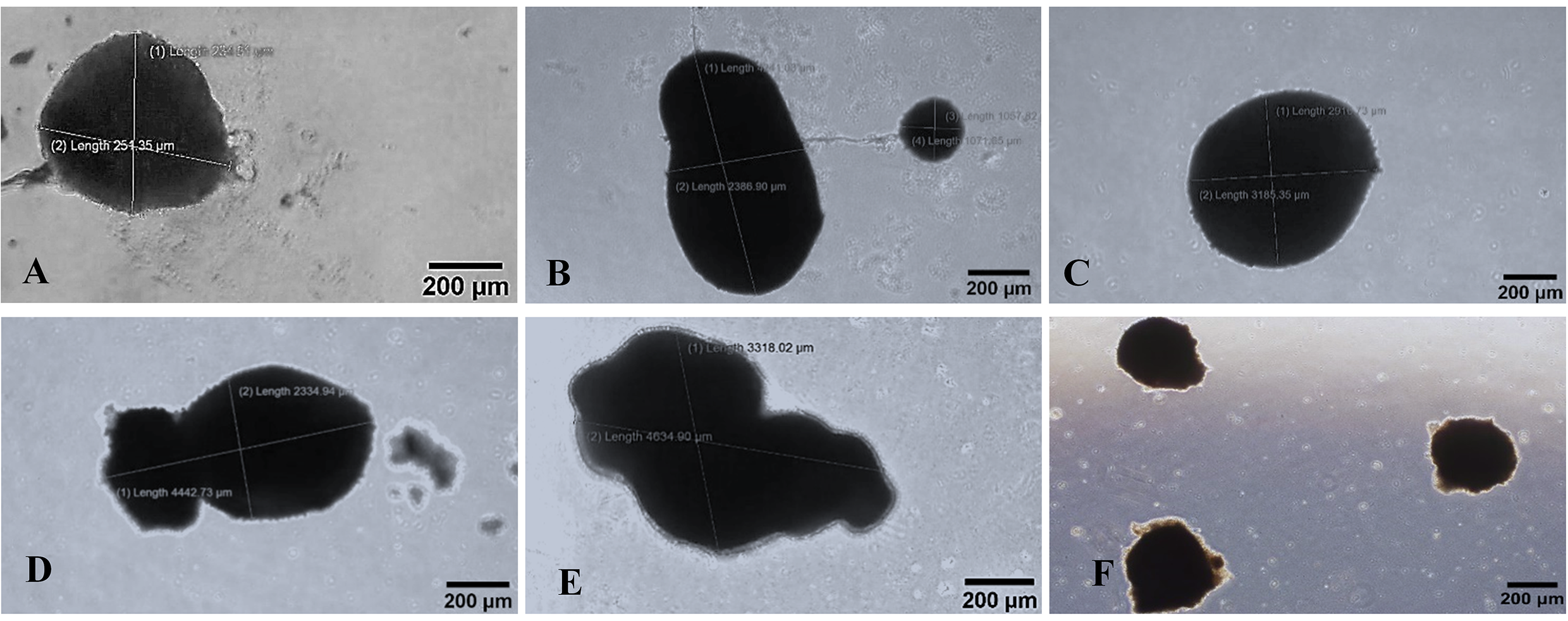

Supplement: Supplementary file 2 — Figure S2. [file JCMM-28-e18453-s002.tif]
